# Correction: Surface modification of carbon cloth anodes for microbial fuel cells using atmospheric-pressure plasma jet processed reduced graphene oxides

**DOI:** 10.1039/c8ra90008f

**Published:** 2018-02-15

**Authors:** Shih-Hang Chang, Bo-Yen Huang, Ting-Hao Wan, Jian-Zhang Chen, Bor-Yann Chen

**Affiliations:** Department of Chemical and Materials Engineering, National I-Lan University I-Lan 260 Taiwan shchang@niu.edu.tw; Graduate Institute of Applied Mechanics, National Taiwan University Taipei City 10617 Taiwan jchen@ntu.edu.tw

## Abstract

Correction for ‘Surface modification of carbon cloth anodes for microbial fuel cells using atmospheric-pressure plasma jet processed reduced graphene oxides’ by Shih-Hang Chang *et al.*, *RSC Adv.*, 2017, **7**, 56433–56439.

The authors regret that an incorrect grant number for B. Y. Chen was given in the original manuscript. The correct acknowledgements section is as shown below.

The authors gratefully acknowledge the financial support provided by the Ministry of Science and Technology (MOST), Taiwan, under Grant No. MOST 104-2221-E-197-004-MY3 (S. H. Chang), 105-2221-E-002-047-MY3 (J. Z. Chen) and 106-2221-E-197-020-MY3 (B. Y. Chen).

The Royal Society of Chemistry apologises for these errors and any consequent inconvenience to authors and readers.

## Supplementary Material

